# Neutralizing antibodies against SARS-CoV-2 variants following mRNA booster vaccination in adults older than 65 years

**DOI:** 10.1038/s41598-022-24409-w

**Published:** 2022-11-27

**Authors:** Christine Durier, Laetitia Ninove, Maeva Lefebvre, Anne Radenne, Corinne Desaint, Jacques Ropers, Rebecca Bauer, Said Lebbah, Diane Carette, Marie Lachatre, Anne-Sophie Lecompte, Dominique Deplanque, Elisabeth Botelho-Nevers, Anne Conrad, Bertrand Dussol, Zoha Maakaroun-Vermesse, Giovanna Melica, Jean-François Nicolas, Renaud Verdon, Jacques Kiladjian, Paul Loubet, Catherine Schmidt-Mutter, Christian Dualé, Séverine Ansart, Stéphane Priet, Axel Levier, Diana Molino, Louis-Victorien Vieillard, Béatrice Parfait, Jean-Daniel Lelièvre, Eric Tartour, Xavier de Lamballerie, Odile Launay, ANRS0002S CoviCompareP Group, Gilbert Mchantaf, Berthe-Marie Imbert-Marcille, Samantha Montagne, Bijan Ghaleh-Marzban, Alexandra Traverse-Glehen, Denis Vivien, Bruno Cassinat, Alexandre Evrard, Catherine Metzger, Jean-Marc Lessinger, Michel Billing, Marc Berger, Christophe Leroyer, Eric Tartour, Victor Appay, Frédéric Batteux, Béhazine Combadière, Guy Gorochov, Stéphane Paul, Sylvie Van Der Werf, Christiane S. Eberhardt, Claire-Anne Siegrist, Michel Cogné, Isabelle Pellegrin, Marc Eloit, Emmanuelle Netzer, Martine Resch, Marine Saouzanet, Laurence Meyer, Marion Bonneton, Raphaelle Tardieu, Soizic Le Mestre, Solange Jancrey-Laval, Ventzislava Petrov-Sanchez, Eric Rosenthal, Yazdan Yazdanpanah, Daniel Olive, Raphaelle Tardieu, Ventzislava Petrov-Sanchez, Amel Bouakane, Veronique Rieux, Claire Madelaine, Soizic Lemestre, Alpha Diallo, Solange Lancrey-Javal, Laurence Meyer, Emmanuelle Netzer, Marine Saouzanet, AP-HP CoviCompareM Group, Florent Valour, Bertrand Dussol, Giovanna Melica, Biological resource centers, Gilbert Mchantaf, Michael Hisbergues, Frédérique Bertholon, Vinca Icard, Annachiara de Sandre-Giovannoli, Bijan Ghaleh-Marzban, Laboratories, Victor Appay, Frédéric Batteux, Béhazine Combadière, Guy Gorochov, Stéphane Paul, Sylvie Van Der Werf, Christiane S. Eberhardt, Claire-Anne Siegrist, Michel Cogné, Trial coordination, Margot Dropy, Fatiha Bouchama, Mehdi Thamri, Saïd Lebbah, Marion Bonneton, Sponsor, Shorheh Azimi, Scientific Committee, Beatrice Parfait, Victor Appay, Frédéric Batteux, Béhazine Combadière, Guy Gorochov, Stéphane Paul, Sylvie Van Der Werf, Claire-Anne Siegrist, Michel Cogné, Florent Valour

**Affiliations:** 1 INSERM US19, Villejuif, France; 2 Unité des Virus Émergents (UVE), Aix Marseille Univ, IRD 190, INSERM 1207, Marseille, France; 3 Maladies Infectieuses et Tropicales, Centre de Prévention des Maladies Infectieuses et Transmissibles CHU de Nantes, INSERM CIC1413, Nantes, France; 4 Assistance Publique Hôpitaux de Paris, Unité de Recherche Clinique des Hôpitaux Universitaires Pitié Salpêtrière –Hôpitaux Universitaires Pitié Salpêtrière - Charles Foix, Paris, France; 5 INSERM CIC 1417 Cochin Pasteur, Assistance Publique Hôpitaux de Paris, Hôpital Cochin, Innovative Clinical Research Network in Vaccinology, Université de Paris, Sorbonne Paris Cité, Paris, France; 6 INSERM, CHU Lille, CIC 1403 - Centre d’investigation Clinique, Univ. Lille, Lille, France; 7 Infectious Diseases Department, CIC 1408 INSERM University Hospital of Saint-Etienne, University Hospital of Saint-Etienne, Saint-Etienne, France; 8 Department Maladies Infectieuses et Tropicales, Hôpital de la Croix-Rousse, Hospices Civils de Lyon, Univ. Claude Bernard Lyon I, CNRS, UMR5308, ENS de Lyon, Univ Lyon, Lyon, France; 9 Centre d’Investigation Clinique 1409, Hôpital de la Conception, INSERM- Hôpitaux Universitaires de Marseille – Aix Marseille Université, Marseille, France; 10 Centre de Vaccination CHU de Tours, Centre d’Investigation Clinique CIC 1415, INSERM, CHRU de Tours, Tours, France; 11 Service d’Immunologie Clinique et Maladies Infectieuses, APHP, Hôpital Henri Mondor, Créteil, France; 12 Centre d’Investigation Clinique 1430 INSERM, APHP, Hôpital Henri Mondor, Créteil, France; 13 Centre International de Recherche en Infectiologie (CIRI), INSERM U1111, Université Claude Bernard Lyon I, Lyon, France; 14 CHU Lyon-Sud, Pierre-Bénite, France; 15 Service de Maladies Infectieuses, CHU de Caen, Caen, France; 16 Dynamicure INSERM, UMR 1311, Normandie Univ, UNICAEN, Caen, France; 17 AP-HP, Hôpital Saint-Louis, Centre d’Investigations Cliniques, INSERM, CIC1427, Université Paris Cité, Paris, France; 18 VBMI, INSERM U1047, Department of Infectious and Tropical Diseases, Université de Montpellier, CHU Nîmes, Univ Montpellier, Nîmes, France; 19 Inserm CIC 1434, CHU Strasbourg, Strasbourg, France; 20 Centre d’Investigation Clinique (INSERM CIC1405), CHU Clermont-Ferrand, Clermont-Ferrand, France; 21 Centre d’Investigation Clinique CIC 1412, INSERM, CHU Brest, Brest, France; 22 ANRS | Emerging Infectious Diseases, Paris, France; 23 Fédération des Centres de Ressources Biologiques - Plateforme de Ressources Biologiques APHP -Université de Paris, Centre de Ressources Biologiques Cochin, Hôpital Cochin, Paris, France; 24 INSERM U955, Vaccine Research Institute, Créteil, France; 25 APHP, Hôpital Européen Georges Pompidou, INSERM U970, PARCC, Université de Paris, Paris, France; 26 APHP, Direction de la Recherche Clinique et de l’innovation (DRCI), Paris, France; 27 CRB Site Cochin, AP-HP, Hôpital Cochin, Paris, France; 28 Laboratoire de Virologie, CHU Nantes, Nantes, France; 29 CRB du CHRU de Tours, Tours, France; 30 PRB, AP-HP, Hôpital Henri Mondor, Créteil, France; 31 HCL, CRB Lyon Sud, Lyon, France; 32 CRB InnovaBIO, CHU Caen Normandie, Caen, France; 33 Service de Biologie Cellulaire, AP-HP, Hôpital Saint Louis, Paris, France; 34 CRB du CHU de Nîmes, Nîmes, France; 35 Unité de Coordination de la Biologie des Essais Cliniques, HUS, Nouvel Hôpital Civil, Strasbourg, France; 36 Laboratoire de Biochimie et Biologie Moléculaire, HUS, Nouvel Hôpital Civil, Strasbourg, France; 37 CRB Auvergne, Hôpital Estaing, CHU Clermont-Ferrand, Clermont-Ferrand, France; 38 CRB Site CIC INSERM CIC1412, Hôpital de la Cavale Blanche, CHRU Brest, Brest, France; 39 INSERM CNRS UMR 5164, Université de Bordeaux, Bordeaux, France; 40 Immunologie Biologique, Hôpital Cochin, INSERM U1016, CNRS UMR 8104, Université de Paris, Paris, France; 41 INSERM U1135, Centre d’Immunologie et des Maladies Infectieuses Sorbonne Université, Paris, France; 42 CIC 1408 INSERM/ANRS, Faculté de Médecine, CHU Saint-Etienne, Saint-Priest En Jarez, France; 43 Centre National de Référence des Virus Respiratoires, Université de Paris Institut Pasteur, Paris, France; 44 Centre de Vaccinologie, Hôpitaux Universitaires de Genève, Suisse, Geneve, Switzerland; 45 INSERM U1236 B Cell Ig Remodeling Singularities (BIGRES) Faculty of Medicine, French Blood Center (EFS Bretagne), University Hospital, Rennes, France; 46 Service d’Immunologie et Immunogénétique, CHU Bordeaux, Bordeaux, France; 47 Pathogen Discovery Laboratory, Institut Pasteur, Paris, France; 48 Centre de Recherche en Cancérologie de Marseille, Marseille, France; 49 CRB, CHRU Lille, Lille, France; 50 CHU Saint-Etienne, CRB42-BTK Saint Priest en Jarez, France; 51 HCL, CRB Lyon Nord, Lyon, France; 52 CRB-APHM, APHM, Hôpital la Timone Enfants, Marseille, France

**Keywords:** Biomarkers, Medical research

## Abstract

Immune response induced by COVID-19 vaccine booster against delta and omicron variants was assessed in 65 adults (65–84 years old) early aftesr a first booster dose. An increase in SARS-CoV-2 neutralizing antibodies was shown in individuals not previously infected without evidence of an age-related effect, with lower increase in those infected before a single dose of primary vaccination. Of note, humoral response was observed only starting from the 5th day after the boost.

## Introduction

Numerous vaccines were developed in an emergency to control the COVID-19 pandemic. mRNA vaccines such as BNT162b2 and mRNA-1273 have rapidly shown their efficacy in protecting against SARS-CoV-2 infection, especially against severe forms of the disease, inducing over 90% protection in the early stages of SARS-CoV-2 infection^1,2^.

The initial randomized controlled trials preferentially targeted young populations. However, the determination of the vaccine response in elderly subjects (> 65 years of age) quickly appeared necessary, as advanced age represents both a risk factor for severe forms of COVID-19 and a widely analyzed determinant of poor response to vaccinations^3^. The initial efficacy of these vaccines was challenged by two non-mutually exclusive parameters which are the loss of immunity over time^4,5^ and the emergence of variants of concern characterized by their escape to the antibody response^6^. This has led several countries to modify their vaccination strategy by recommending the use of a third dose of vaccine, which has the capacity to stimulate memory B populations, leading to an increase in the level of circulating antibodies associated with a broadening of their spectrum^7^.

The correlates of protection with COVID-19 vaccines are still under discussion, although the circulating level of neutralizing antibodies seems to be a good marker of protection^8^. Several teams have shown that vaccines are able to induce an effective memory B response, which explains the effect of a booster dose at a distance from the primary vaccination. However, in the context of a decrease in circulating antibody levels, the kinetics of new antibody production in the face of a rapidly replicating virus are not known. Although some studies report a delay in antibody production after booster vaccination of 3 to 5 days on average with other vaccines, this has been in fact poorly studied ^9^.

The CoviCompare research program launched in France in January 2021 aims to assess the immunogenicity of different COVID 19 vaccine platforms in older people (65 years and older) compared to younger people (18–45 years)^10^. In CoviCompareM and CoviCompareP trials (ClinicalTrials.gov: NCT04748471 and NCT04824638), participants with negative SARS-CoV-2 serology at inclusion and no previous history of COVID-19 received two doses of either mRNA-1273 or BNT162b2, 28 days apart, as primary vaccination. Participants with a documented history of SARS-CoV-2 infection were also included in CoviCompareP. In addition, as French vaccine recommendations have included the use of a third vaccine over time, participants were also able to receive an additional dose of vaccine from mid-October 2021.

We present the results of a combined sub-study of these two trials in which eligible participants were those aged 65 years and older who had received a booster dose and whose samples were available in mid-December 2021. We examined SARS-CoV-2-specific neutralizing antibodies for European (D614G), Delta and Omicron (BA.1) variants at the time of boost, and 3, 15, and 28 days post-boost.

Our objectives were to study the early neutralizing response against different variants and to explore the effect of age among older adults on immune response.

## Methods

### Study design and participants

In CoviCompareM (ClinicalTrials.gov NCT04748471) and ANRS002S CoviCompareP (ClinicalTrials.gov NCT04824638) trials, adults not previously infected with SARS CoV-2 (negative SARS-CoV-2 serology and PCR at inclusion and no previous history of COVID-19), received two full doses of either mRNA-1273 or BNT162b2 vaccine, 28 days apart. In CovicompareP trial, were also included participants with documented history of SARS-CoV-2 infection at least 5 months before the inclusion. These patients received a single dose of BNT162b2 vaccine according to the French COVID-19 immunization guidelines at the time. Participants were healthy adults or with stable medical condition (defined as disease not requiring change in therapy or hospitalization for worsening disease during 3 months before enrolment nor expected significant change in foreseeable future)^10^.

In accordance with French government decisions, a booster dose (second dose for previously infected participants, third dose for the others) was proposed 6 months after the primary vaccination, initially to participants aged 65 years and older. For BNT162b2, a full dose (30 µg) while for mRNA-1273, a half dose (50 µg) was administered. The protocols and all amendments of this combined sub-study had the approval of the Ethics Committee (CPP of Ile de France 1) and the national drug regulatory authority (ANSM). Signed informed consent was obtained from each participant. All methods were performed in accordance with the relevant guidelines and regulations.

In this study, we included participants aged 65 years or older, who had no SARS-CoV-2 infection during the study (documented infectious episode or positive anti-N serology) who had received their booster dose and for whom blood samples on days 3 and 15 post-boost were available before December 16th 2021.

### Immunogenicity assessments

Serum samples were tested for anti-SARS-CoV-2 IgG antibodies directed against the S1 domain of the spike protein of the virus using a commercial ELISA kit (Euroimmun, Lübeck, Germany) on the day of the third dose, and 3, 15, 28 days after. The kit is based on the Wuhan spike protein. Quantitative results were expressed in standardized units (binding antibody units (BAU) per mL)^11^.

Neutralizing antibodies against the European (D614G), Delta and Omicron (BA.1) variants of SARS-CoV-2 were tested centrally at same timepoints. The B.1 BavPat1 SARS-CoV-2 strain (G614 strain) was obtained from Pr. C. Drosten through EVA GLOBAL (https://www.european-virus-archive.com/) and contains the D614G mutation. Virus stocks of this strain were produced using VeroE6 cells. The clinical strain of the SARS-CoV-2 Delta (B 1.617.2) and BA.1 Omicron (B.1.1.529) variants used here are also available through EVA GLOBAL (ref: 001V-04282, GISAID: EPI_ISL_2838050 and 001V-04436, GISAID: EPI_ISL_7899754, respectively). Virus stocks of these strains was produced using VeroE6/TMPRSS2 cells. All virus stocks were characterized by TCID50 determination and whole-genome sequencing (Ion Torrent) in order to verify the absence of additional mutations, especially in the spike-coding region. All experiments with infectious viruses were performed in a biosafety level 3 laboratory. Neutralizing antibodies were also tested one month after the primary vaccination for European variant and for Delta and Omicron variants for a random subsample only (N = 24). We used an in-house microneutralization test as described elsewhere^12^ with a positive seroneutralization defined as a titer ≥ 20.

### Statistical methods

For each variant (European, delta, omicron), the statistical analysis compared the neutralizing antibodies titers between groups and timepoints by applying a longitudinal model (mixed model for repeated measures [MMRM]) accounting for the correlation among repeated measures and adjusted for age. Models for log transformed titers included the fixed effects of group, timepoint and the interaction between group and timepoint with age at inclusion (years) as an additional fixed factor. Within-subject variability was estimated with an unstructured covariance matrix. Pairwise comparisons between groups at the different timepoints and comparisons between timepoints for each group were obtained. A ‘M vs P’ p-value was interpreted when the overall group effect at a timepoint was significant (p ≤ 0.05). Geometric mean titers (GMT) were estimated using standard log transformation for antibody titers and taking the anti-log of the resulting estimates. This approach was followed for the least squares means, least squares means differences and the corresponding two-sided 95% confidence intervals. In order to study the very early post-boost response, as 3–4 days are required in general to generate antibody titers above the protective threshold^9^, post hoc analyses were performed with the same MMRM model excluding participants who had their day 3 evaluation 5 or 6 days after the boost. Geometric means of the post-boost ratios were compared between participants tested on day 5 to 6 and participants tested on day 2 to 4 of the M and P groups. Neutralizing antibody levels were analyzed graphically against the age of the participants at first vaccination dose and using Spearman correlations. Paired signed rank test was also used to compare immune responses obtained 15 days after the boost to 28 days after the primary vaccination. All tests are two-sided. Statistical analyses were conducted using SAS v9.4.

## Results

Sixty-five participants aged 65 years or older were included in the current sub-study, distributed in three groups: participants without previous SARS CoV-2 infection who received two doses plus a booster of mRNA-1273 vaccine (CoviCompareM, referred to in this document as group ‘M’, n = 34), or two doses plus a booster of BNT162b2 (CoviCompareP group 1, or group ‘P’, n = 19) and participants previously infected with SARS-CoV-2 who received a single dose plus a booster of BNT162b2 (CoviCompareP group 2, or group ‘P2’, n = 12).

The median age of the participants was 71 years (IQR: 68–76); 55% (n = 36) were men (Supplementary Table 1). Among participants without previous SARS CoV-2 infection, there were no differences in age, sex, body mass index between those who received mRNA-1273 (M group) or BNT162b2 (P group). The delay between infection and primary vaccination was 11.5 months in median (IQR: 9.5–12.4, range:7.7 to 12.8 months) in subjects with a history of infection (P2 group).

Time between the second dose of the primary vaccination and the boost was significantly longer for mRNA-1273 as compared to BNT162b2 recipients (7.4 and 6.8 months respectively, p < 0.001) with a difference in medians estimated at 19 days. Participants with a history of SARS-CoV-2 infection received the booster dose (second dose) in a median time of 7.8 months after the first dose of BNT162b2.

**Figure 1:**
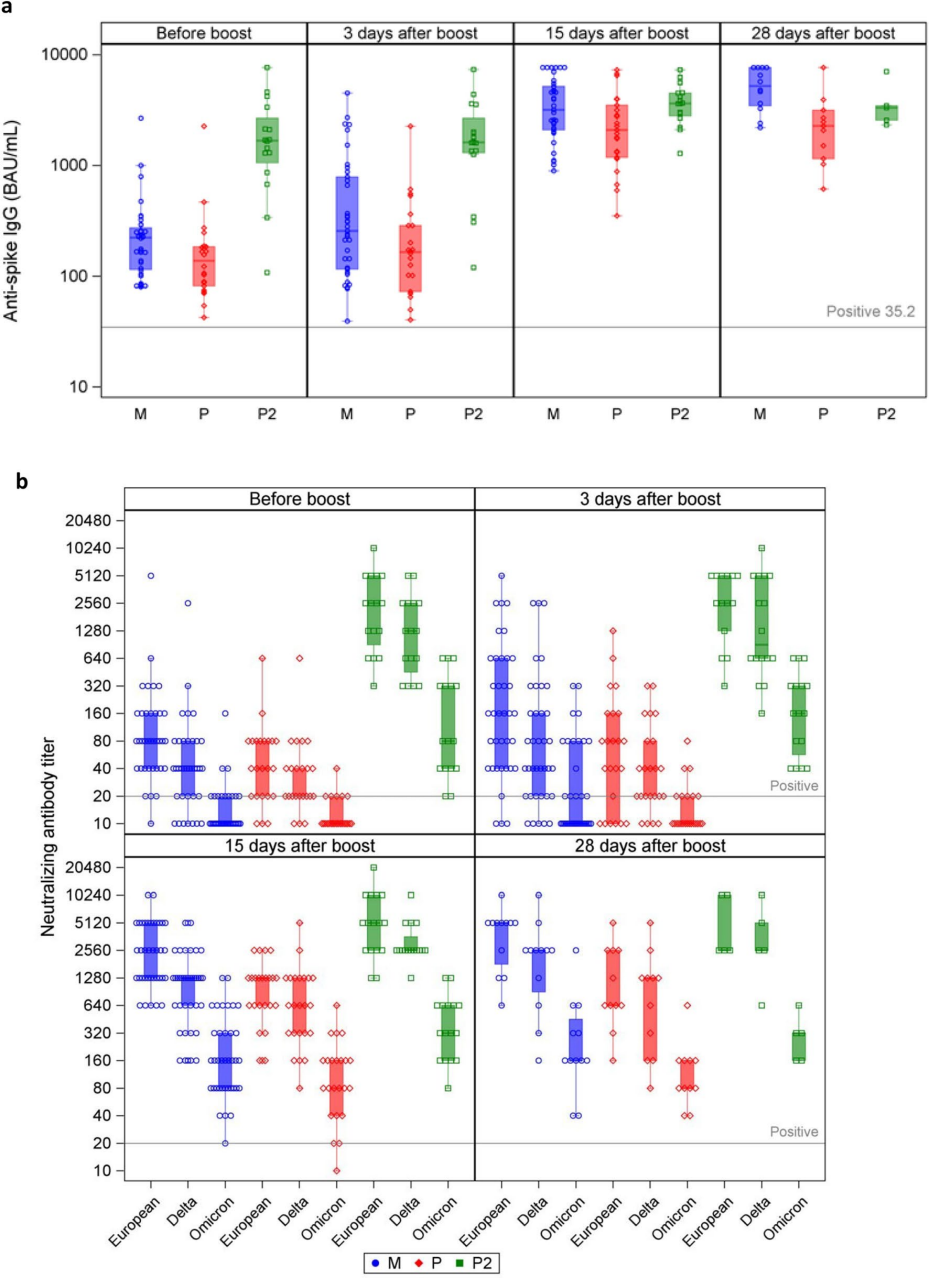

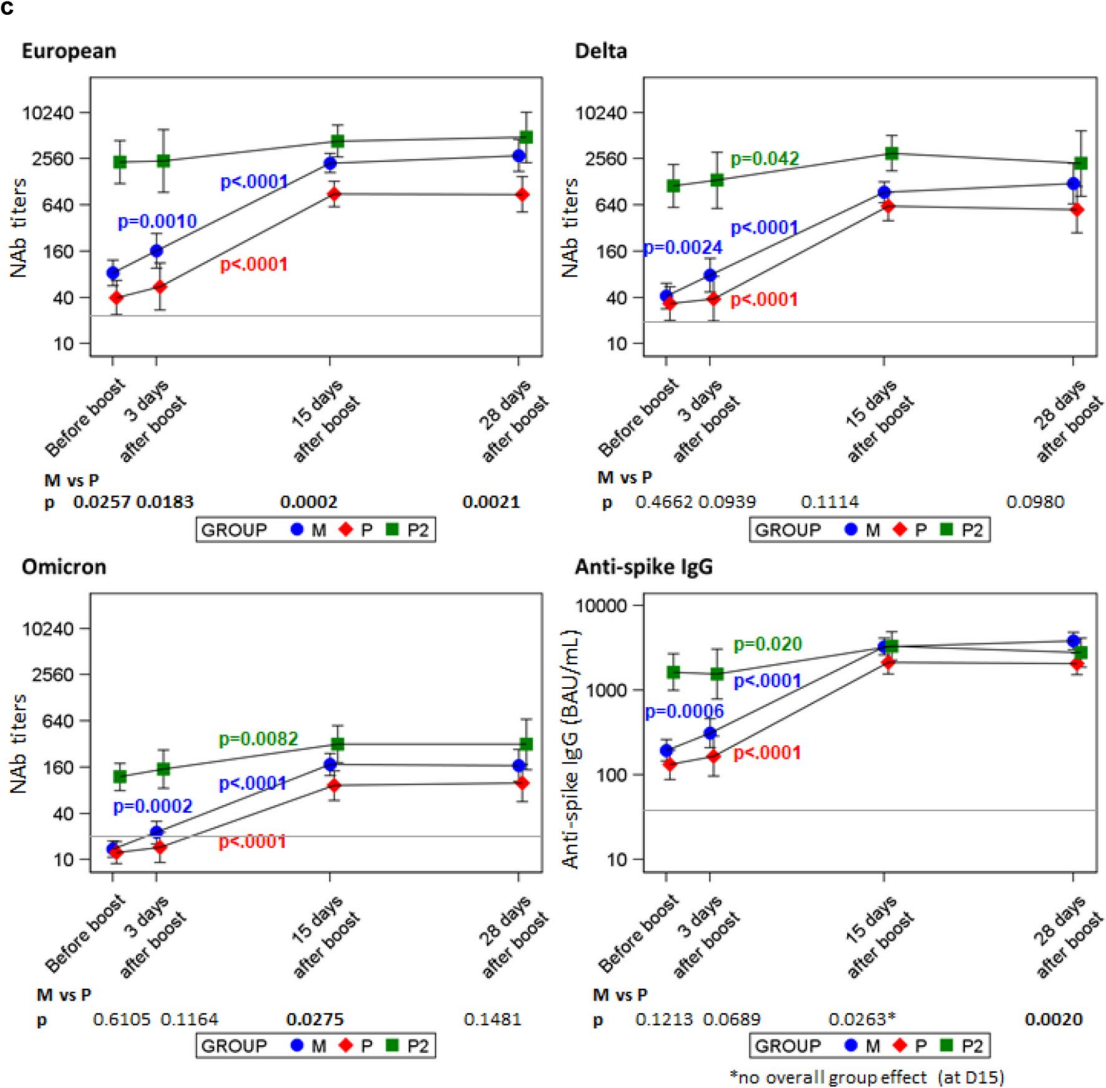
Humoral immune responses in SARS-CoV-2 naïve mRNA-1273 recipients (M in blue, n = 34), BNT162b2 recipients (P in red, n = 19) and previously SARS-CoV-2 infected BNT162b2 recipients (P2 in green, n = 12). (**a**) Anti-spike IgG (BAU/mL). (**b**) Neutralizing antibody titers for European strain, Delta, Omicron (BA.1) variants. (**c**) Geometric means (MMRM model estimates, N = 65). Timepoints comparisons: significant p-values within each group are shown (< .05). M vs P comparisons: p-values at each timepoint below the figures. Grey lines show positivity limits (IgG > 35.2 BAU/mL, titer ≥ 20).

Anti-Spike IgG titers were positive (> 35.2 BAU/mL) in 100% of participants in all groups before and after the boost (Fig. 1a).

Before the booster dose for participants with no previous SARS-CoV-2 infection, neutralizing antibodies were detected—respectively in mRNA1273 (M group) and BNT162b2 (P group) recipients—in 97% (32/33, 1 missing) and 84% (16/19) for the European strain; in 79% (26/33) and 84% (16/19) for Delta variant; and only in 33% (11/33) and 26% (5/19) for Omicron BA.1 variant (Fig. 1b). All participants previously infected with SARS-CoV-2 had detectable neutralizing antibodies for all viral strains before the booster dose.

Neutralizing antibodies reached 100% positivity on day 15 for all groups and all SARS-CoV-2 strains, except for one individual of the P group for Omicron BA.1 variant. Although positivity was achieved at day 15 post-boost for all but one patient, GMT analysis revealed a large disparity between strains (Supplementary Table 2).

**Figure 2:**
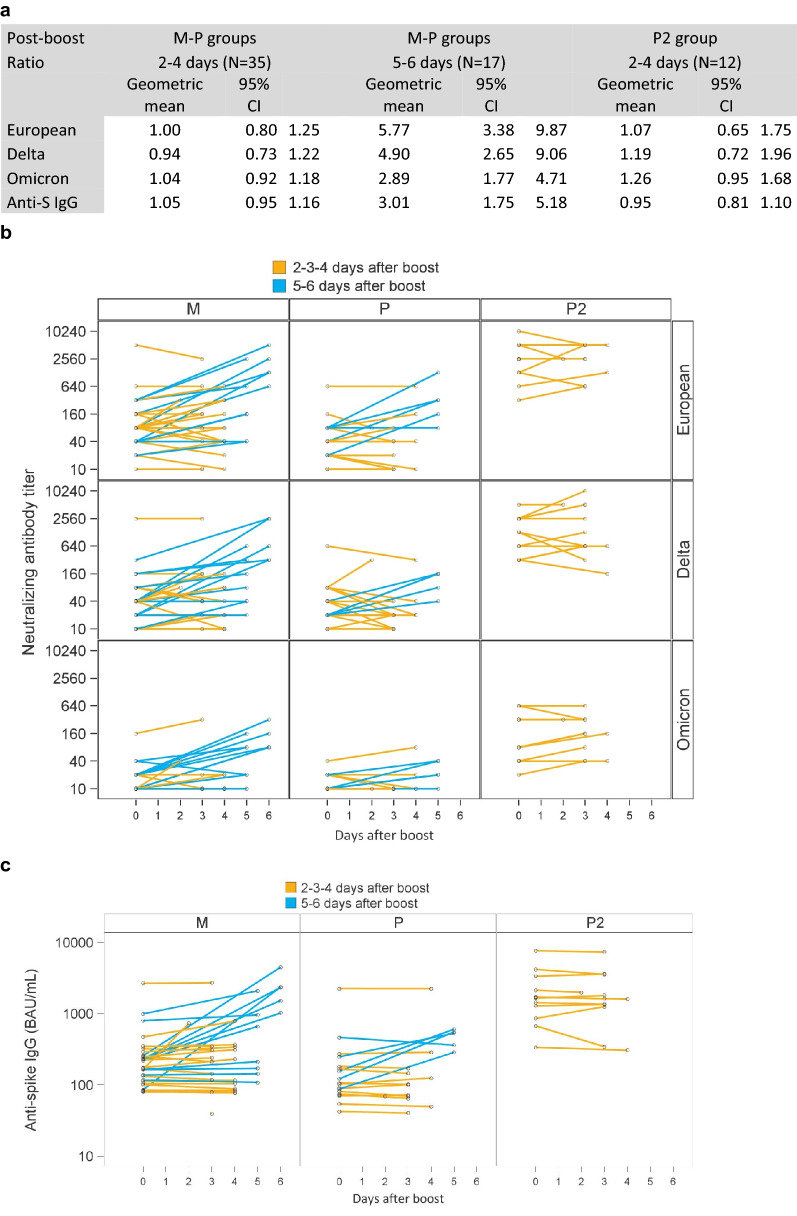
Focus on early antibodies up to day 6 after boost in SARS-CoV-2 naïve mRNA-1273 recipients (M), BNT162b2 recipients (P) and previously SARS-CoV-2 infected BNT162b2 recipients (P2). (**a**) Post boost/pre-boost ratios (geometric means and 95% confidence intervals) (**b**) Neutralizing antibody titers for European strain, Delta, Omicron (BA.1) variants and (**c**) anti-spike IgG (BAU/mL). N = 64/65 (one participant from group M missing pre-boost sample).

Early blood samples allowed us to analyze the kinetics of antibody appearance. The early increase from boost to “day 3” was significant only for M group when considering all available data (Fig. 1c). However, in sensitivity analyses excluding 17/65 participants with samples performed at day 5 or 6, this early change was not significant any more (Supplementary Fig. 1). Moreover, focusing on early data showed that the post-boost ratios of antibody titers for participants tested on day 5 or 6 were higher, and in all groups (M, P and P2) than for participants tested on day 2 to 4 (Fig. 2).

The increase from day 3 to day 15 was significant for all groups, for the European strain and both Delta and Omicron BA.1 variants. All titers were quite stable between day 15 and day 28 and there was no significant change between day 15 and day 28 in any of the groups and for any SARS-CoV-2 variant (Fig. 1c). A higher immune response was induced with mRNA-1273 than BNT162b2 for the European strain (day 0 p = 0.0257, day 3 p = 0.0183, day 15 p = 0.0002, day 28 p = 0.0021), but the difference was not significant for the Delta and Omicron BA.1 variants (except at day 15 for Omicron BA.1 p = 0.0275).

No significant correlation between age at the first dose and antibody titers was observed (Supplementary Fig. 2). Neutralizing antibodies against European, Delta and Omicron BA.1 available for sub samples of participants one month after the second dose of primary vaccination were significantly lower to levels obtained 15 days after the boost (Supplementary Fig. 3).

## Discussion

The results of our study confirm those obtained by others showing the immunological benefit of a third dose of mRNA vaccine against SARS-CoV2 in terms of induction of a neutralizing antibody response. We also note that if this benefit exists for the induction of a response against all viral strains, it appears more limited against Omicron, the antibody titer remaining much lower than that obtained against the European and Delta strains.

The immune response in the elderly has so far been little explored and a decrease in neutralization capacity has been shown for primary vaccination in vaccinees over 80 years of age^7^. Up to one month after the booster dose, we did not find evidence of an age effect between 65 and 84 years. This parallels efficacy data from Israel, where the efficacy of the third dose of the vaccine against hospital admission and severe illness was found to be similar between those aged 40–69 years and those aged 70 years or older^8^.

A significantly higher humoral immunogenicity of the mRNA-1273 vaccine compared with the BNT162b2 vaccine across age categories has been shown^13^. For homologous boosters, we also showed higher neutralizing antibodies against the European strain with a half dose of mRNA-1273 as compared to BNT162b2.

A second late mRNA vaccine dose in persons with a history of infection has a minimal immunological effect and not always significant^14,15^. Here, we showed that early-infected subjects who received a single dose of vaccine an average of 7.8 months prior had significant neutralizing antibody levels against all viral strains. Although significant, a booster dose induced only a moderate increase in this antibody level, questioning the relevance of the booster. Indeed, if such a recall is likely to reduce the occurrence of new infections in this population, it does not modify the impact on the protection against severe forms, which remains high^16,17^. On the other hand, it is important to keep in mind that the immunological implications of this hybrid immunity are to be analyzed with regard to the strain that led to the initial infection.

In non-previously infected participants, levels of neutralizing antibodies exceeded after the boost (day 15, 28) those observed after the primary vaccination as already reported^18,19^ and 100% of positive neutralizing antibodies against Delta and Omicron variants were obtained at day 15 and 28^20–22^.

Finally, an important point of our study was to be able to study the kinetics of neutralizing antibody appearance after a third dose of vaccine. Interestingly, early response to a booster was evidenced against all variants only after the fifth day. It was recently shown in a SARS-CoV-2 human challenge study that the wild-type virus begins to replicate as early as the 40th hour post-inoculation in the throat (1.7 days) with a peak of replication in the nose on Day 6^23^. Because of this early replication and the delay of response to the booster shown here, maintaining high titers of neutralizing antibodies appears to be crucial for protection, and in favour of booster doses, especially for elderly.

The weakening of humoral immunity in the elderly population^24^ suggests that further follow-up data from the CoviCompare trials will help to understand how humoral but also cellular responses evolve over the longer term.

## Supplementary Information

Supplementary Information.

## Data Availability

As these trials are ongoing, access to the subject level data presented here will be available upon request once the trials are completed, subject to legal provisions and the signing of an appropriate contract. Please contact corresponding author for data requests.
