# Supplementary material for: Neutralizing antibodies against SARS-CoV-2 variants following mRNA booster vaccination in adults older than 65 years
Source: Sci Rep. 2022 Nov 27;12:20373. doi: 10.1038/s41598-022-24409-w (PMC9701678; doi:10.1038/s41598-022-24409-w)

Supplementary Table 1: Characteristics of the population

	SARS-CoV-2 naïve			SARS-CoV-2 infected before the primary vaccination
	CoviCompareM (M) mRNA-1273 N = 34	CoviCompareP (P) BNT162b2 N = 19	p-value	CoviCompareP (P2) BNT162b2 N = 12
Age (years)	71.5 [68.2, 76.0] (66 to 84)	72.0 [66.5, 75.5] (65 to 84)	0.56[1]	70.0 [67.0, 75.0] (65 to 81)
Male sex	20 (59%)	8 (42%)	0.24[2]	8 (67%)
Body Mass Index (BMI)	25.3 [22.6, 27.6]	24.7 [22.3, 26.6]	0.48[1]	24.0 [20.7, 25.0]
BMI > 30	5 (15%)	0 (0%)	0.15[3]	0 (0%)
Months between primary vaccination and boost	7.4 [7.0, 7.5]	6.8 [6.4, 7.0]	<0.001[1]	7.8 [7.5, 7.9]
3 days after boost, *days*	4 (2 to 6)	3 (2 to 5)	0.12[1]	3 (2 to 4)
15 days after boost, *days*	15 (12 to 17)	14 (13 to 20)	0.25[1]	14 (14 to 17)
28 days after boost, n	12	11		5
days	28 (26 to 29)	28 (25 to 28)	0.26[1]	28 (27 to 28)

n (%); Median [IQR] (min to max)
[1]Wilcoxon rank sum test [2] Pearson's Chi-squared test [3]Fisher's exact test

Supplementary Table 2: **Geometric means (MMRM model estimates)**

Individual profiles are connected with grey lines and model-based geometric means estimates with 95% confidence intervals are connected with black lines at Day 0=before boost, 3, 15, 28 days after boost

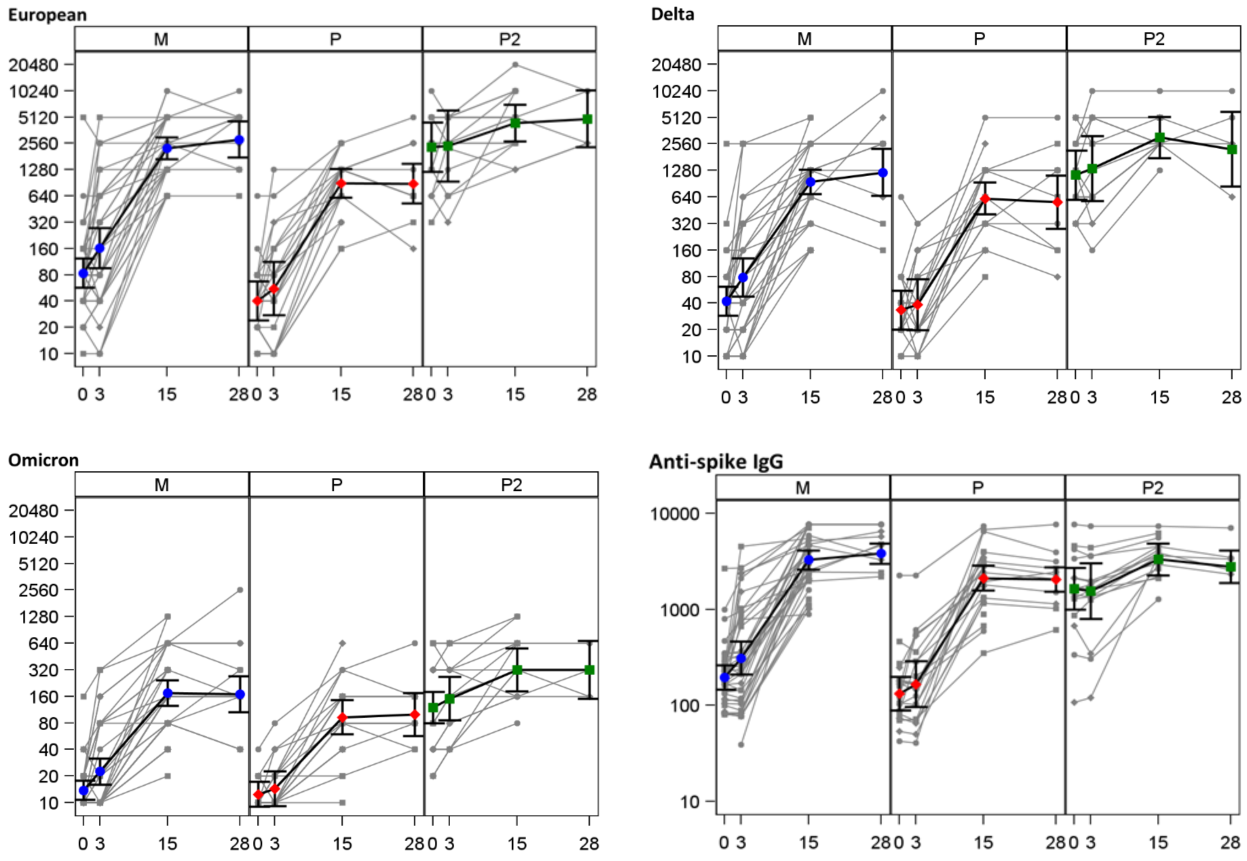

European strain (neutralization titer)

Timepoint	CoviCompare-M mRNA-1273 SARS-CoV-2 naïve (M) N=34	CoviCompare-P BNT162b2 SARS-CoV-2 naïve (P) N=19	CoviCompare-P BNT162b2 previously SARS CoV-2 infected (P2) N=12
Before boost (Day 0), n	33	18	12
**Geometric mean**	**83.63**	**40.08**	**2317.65**
95%CI	56.79, 123.17	23.99, 66.96	1212.41, 4430.42
Day 3, n	34	18	12
**Geometric mean**	**162.30**	**55.66**	**2392.06**
95%CI	95.68, 275.32	27.46, 112.81	937.77, 6101.66
Day 15, n	34	19	12
**Geometric mean**	**2251.43**	**890.57**	**4375.14**
95%CI	1692.60, 2994.74	608.61, 1303.14	2701.65, 7085.25
Day 28, n	12	11	5
**Geometric mean**	**2828.78**	**883.94**	**4878.70**
95%CI	1753.91, 4562.38	523.47, 1492.62	2302.26, 10338.41

Delta variant (neutralization titer)

Timepoint	CoviCompare-M mRNA-1273 SARS-CoV-2 naïve (M) N=34	CoviCompare-P BNT162b2 SARS-CoV-2 naïve (P) N=19	CoviCompare-P BNT162b2 previously SARS CoV-2 infected (P2) N=12
Before boost (Day 0), n	33	19	12
**Geometric mean**	**42.06**	**33.31**	**1135.41**
95%CI	28.68, 61.70	20.06, 55.32	598.24, 2154.94
Day 3, n	34	19	12
**Geometric mean**	**78.52**	**38.55**	**1350.24**
95%CI	47.59, 129.56	19.74, 75.27	580.60, 3140.14
Day 15, n	34	19	12
**Geometric mean**	**944.41**	**616.76**	**3031.19**
95%CI	688.45, 1295.52	404.55, 940.31	1777.21, 5169.96
Day 28, n	12	12	5
**Geometric mean**	**1217.55**	**558.93**	**2228.93**
95%CI	659.30, 2248.47	277.66, 1125.13	838.54, 5924.74

Omicron BA.1 variant (neutralization titer)

Timepoint	CoviCompare-M mRNA-1273 SARS-CoV-2 naïve (M) N=34	CoviCompare-P BNT162b2 SARS-CoV-2 naïve (P) N=19	CoviCompare-P BNT162b2 previously SARS CoV-2 infected (P2) N=12
Before boost (Day 0), n	33	19	12
**Geometric mean**	**13.82**	**12.45**	**119.95**
95%CI	10.80, 17.69	8.99, 17.24	79.40, 181.23
Day 3, n	34	19	12
**Geometric mean**	**22.60**	**14.40**	**151.13**
95%CI	16.10, 31.70	9.16, 22.64	85.38, 267.51
Day 15, n	34	19	12
**Geometric mean**	**173.54**	**92.58**	**320.23**
95%CI	124.35, 242.20	59.31, 144.51	182.53, 561.82
Day 28, n	12	12	5
**Geometric mean**	**169.49**	**99.78**	**319.78**
95%CI	105.79, 271.56	57.40, 173.44	149.93, 682.03

Anti-spike IgG (BAU/mL)

Timepoint	CoviCompare-M mRNA-1273 SARS-CoV-2 naïve (M) N=34	CoviCompare-P BNT162b2 SARS-CoV-2 naïve (P) N=19	CoviCompare-P BNT162b2 previously SARS CoV-2 infected (P2) N=12
Before boost (Day 0), n	33	18	12
**Geometric mean**	**195.77**	**132.24**	**1637.59**
95%CI	145.60, 263.21	88.46, 197.67	997.00, 2689.76
Day 3, n	34	18	12
**Geometric mean, n**	**311.72**	**166.85**	**1553.70**
95%CI	209.35, 464.13	96.75, 287.74	794.51, 3038.32
Day 15, n	34	19	12
**Geometric mean**	**3272.47**	**2121.77**	**3319.86**
95%CI	2604.99, 4110.96	1565.07, 2876.50	2258.85, 4879.25
Day 28, n	12	11	5
**Geometric mean**	**3813.58**	**2057.77**	**2793.87**
95%CI	3003.65, 4841.90	1532.71, 2762.69	1890.07, 4129.84

Supplementary Figure 1: **Geometric means and 95% Confidence Intervals (MMRM model estimates) for participants with 2-4 days post boost only (n=48)**

Neutralizing antibody titers for European strain, Delta, Omicron (BA.1) variants and anti-spike IgG (BAU/mL) in SARS-CoV-2 naïve mRNA-1273 recipients (M in blue), BNT162b2 recipients (P in red) and previously SARS-CoV-2 infected BNT162b2 recipients (P2 in green) before booster dose (day 0) and at 3, 15 and 28 days after boost.

Timepoints comparisons: significant p-values within each group are shown (<.05)

M vs P comparisons: p-values at each timepoint below the figures

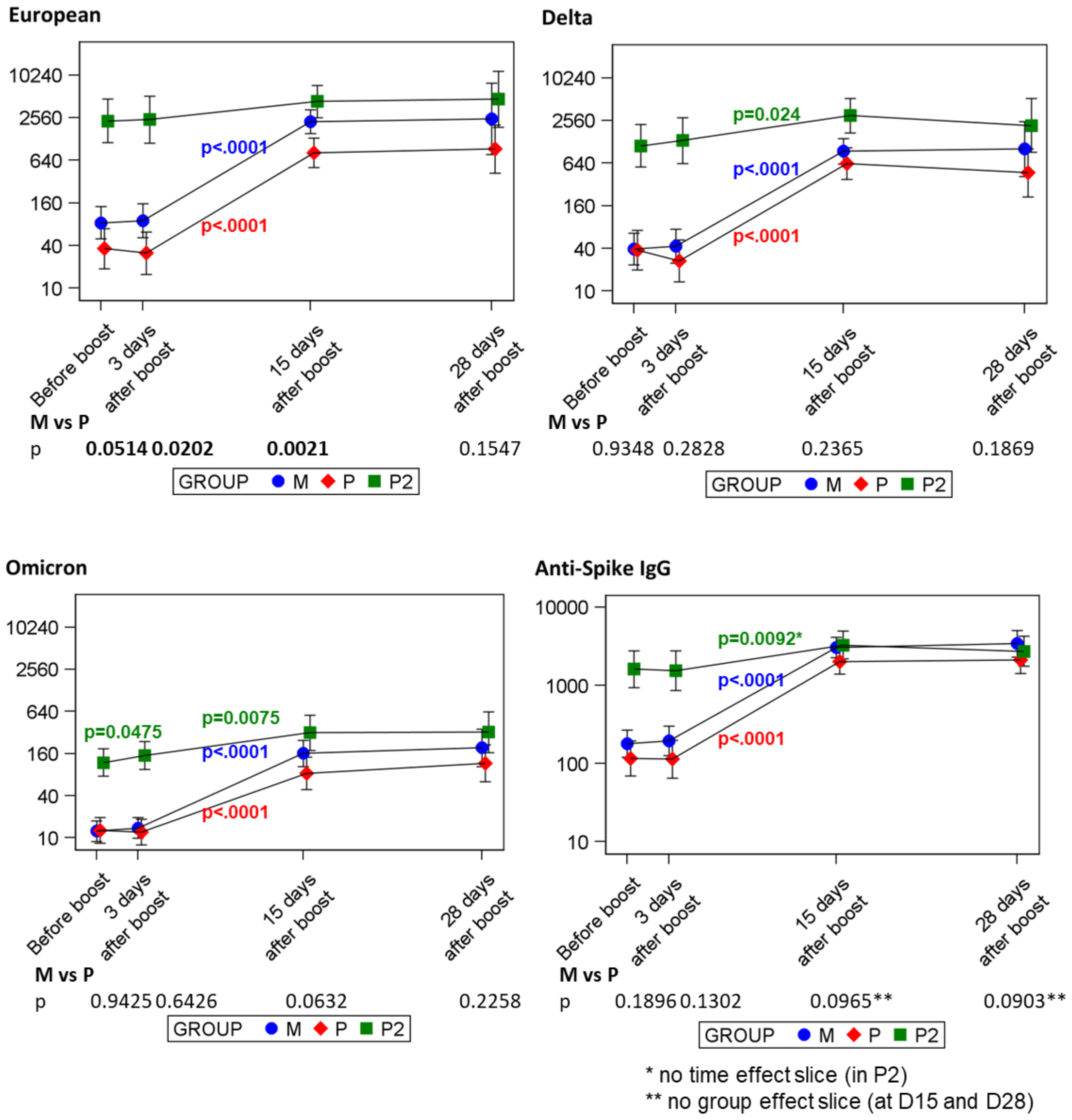

Supplementary Figure 2**: Neutralizing antibody titers for European strain, Delta, Omicron (BA.1) variants against the age at the first dose of vaccine** (years) in SARS-CoV-2 naïve mRNA-1273 recipients (M in blue), BNT162b2 recipients (P in red) and previously SARS-CoV-2 infected BNT162b2 recipients (P2 in green) before booster dose and days 3, 15 and 28 after boost. Regression slopes and Spearman correlations are not statistically significant.

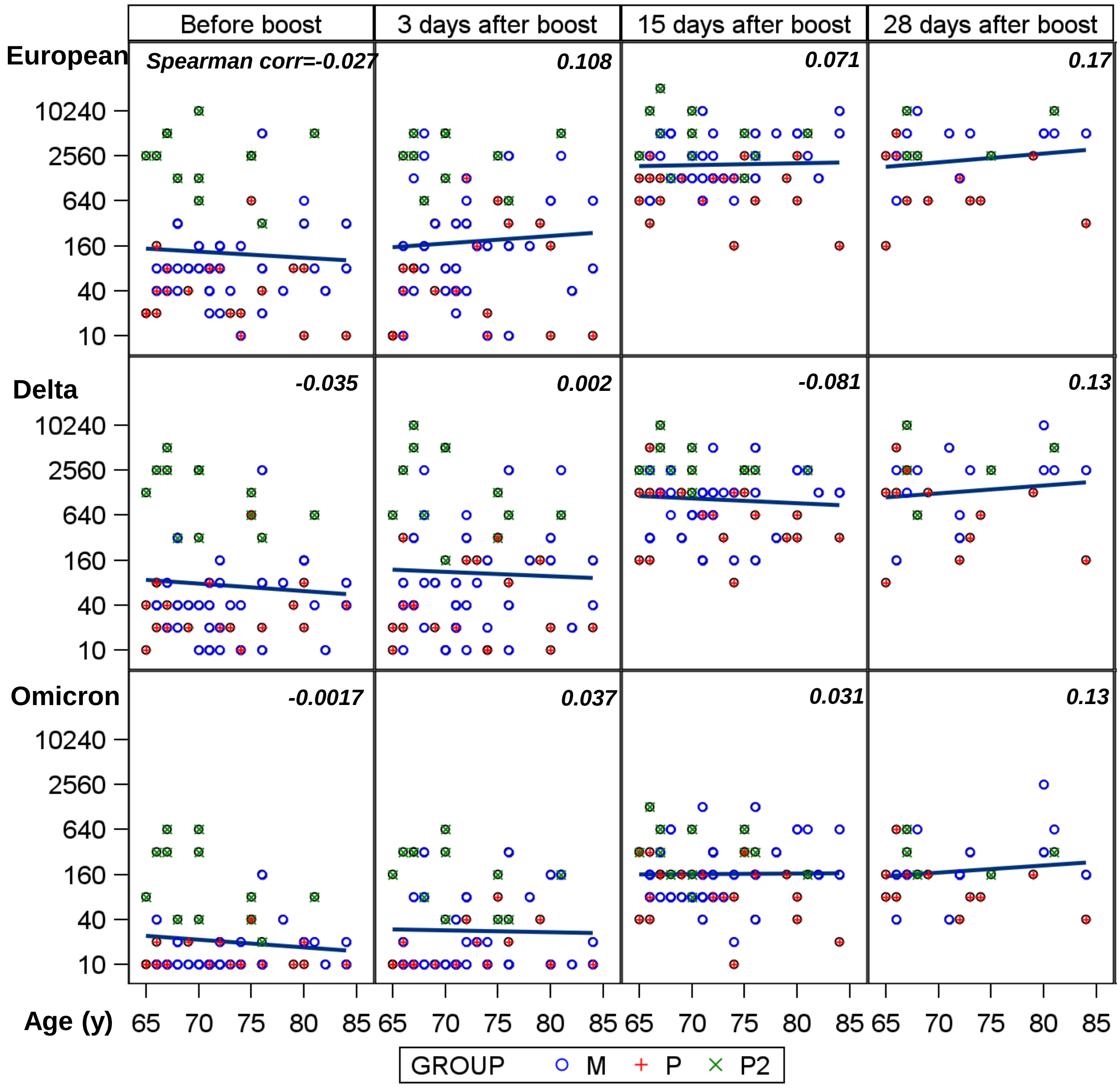

Supplementary Figure 3: Neutralizing antibody titers for European strain, Delta, Omicron (BA.1) variants **at D1 (1st dose) ; D29 (28 days after 1st dose and 2nd dose in SARS-CoV-2 naïve); D57 (28 days after 2nd dose).** SARS-CoV-2 naïve mRNA-1273 recipients (M in blue), BNT162b2 recipients (P in red) and previously SARS-CoV-2 infected BNT162b2 recipients (P2 in green). Sample sizes (N) and medians are indicated at the bottom and p-values are for paired signed rank tests between D15 post-boost and D28 post primary-vaccination when n>5. Black triangles show administration of vaccine.

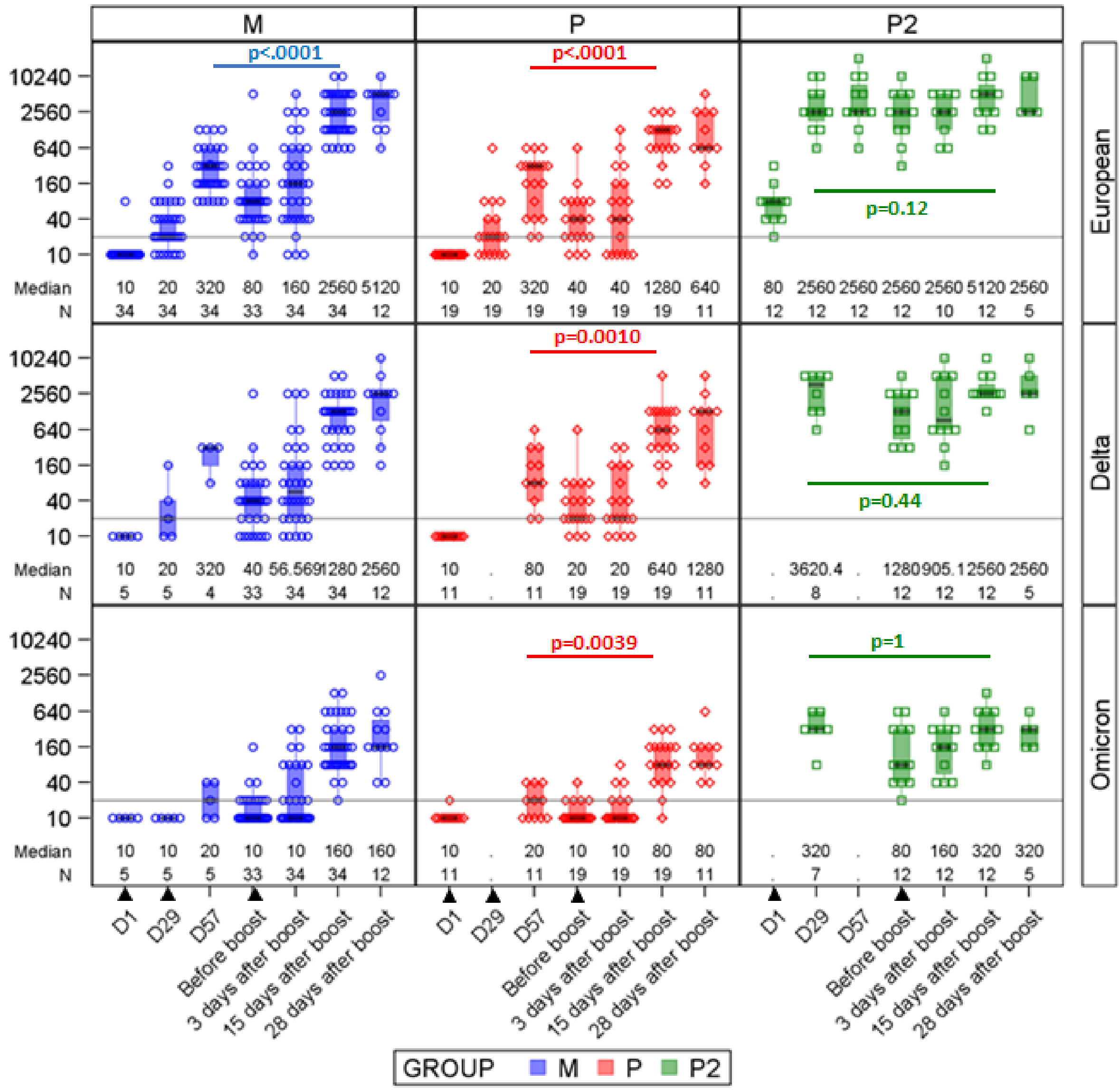

Supplement: Supplementary file 1 — Supplementary Information. [file 41598_2022_24409_MOESM1_ESM.pdf]
